# Time and causality: editorial

**DOI:** 10.3389/fpsyg.2014.00228

**Published:** 2014-03-20

**Authors:** Marc J. Buehner

**Affiliations:** School of Psychology, Cardiff UniversityWales, UK

**Keywords:** time perception, causality, causal inference, temporal adaptation, cognitive development, multimodal integration

It is my great pleasure to be able to introduce the research topic on Time and Causality. The topic had been hosted simultaneously on Frontiers in Perception Science and Frontiers in Cognitive Science. Doing so acknowledged that the human experiences of Time and Causality mutually constrain each other, and attracted high-quality submissions from a wide range of authors who might previously not have published in the same outlet.

The majority of research on Time and Causality in previous decades investigated how temporal information constrains causal inference (for an overview see Buehner, 2005). More specifically, such research is rooted in David Hume's assessment that causal knowledge must be inferred from non-causal input, in a manner where empirical cues of contingency, contiguity, and temporal priority elicit causal impressions in a bottom-up manner (Einhorn and Hogarth, 1986; Buehner and May, 2002). The first half of this volume includes articles from this tradition. Greville and Buehner (2012) pick up on the well-established finding that degrading cause-effect contiguity leads to concomitant decrements in causal learning. Their contribution asked whether the extent to which causal inferences are adversely affected by delay is related to temporal discounting, the phenomenon whereby rewards lose value over time. If causal learning is drawing on principles of associative learning (cf. Dickinson, 2001), then it would be reasonable to find such commonalities; Greville and Buehner (2012), however, do not evidence for such commonalities. Msetfi et al. (2012) revisit a classic phenomenon in covariation-based causal learning: Depressive Realism—the finding that dysphoric individuals appear to have a more realistic impression of the (absence of) cause-effect contingencies. In their contribution, Msetfi et al. (2012) show that dysphoric individuals are particularly sensitive to temporal shifts in contingency, i.e., momentary changes of action-outcome effectiveness.

Rankin and McCormack's (2013) is the first of two developmental articles in the volume and clarifies previously ambiguous or contradictory evidence regarding the understanding of the temporal priority principle—that causes must precede their effects. With improved and standardized methods, Rankin and McCormack (2013) find that even 3 year olds are sensitive to this principle, but also that there is developmental progression toward more consistent application of it. Schlottmann et al.'s (2013) contribution is from the domain of perceptual causality, concerning visual stimuli that lead to immediate and compelling impressions of causality, despite the impoverished nature of the stimuli. Schlottmann et al. (2013) examined the developmental progression of the distinction between physical and social causality, and find that spatio-temporal cues play an important role in making this distinction. Woods et al. (2012) also examined perceptual causality and its sensitivity to spatio-temporal manipulations. They find that context and prior experience heavily influences people's sensitivity to temporal as well as spatial violations of causal expectations.

The second block of articles represents research inspired by relatively recent efforts to examine how causal knowledge influences our perception of time. Temporal binding (Haggard et al., 2002) refers to the subjective shortening of time that occurs when a cause is followed by its effect (as opposed to an unrelated event), and/or subjective shifts in event perception whereby causes and effects mutually attract each other, resulting in delayed awareness of the former, and early awareness of the latter. Faro et al. (2013) open this section with a review of recent literature in this area. Moore et al. (2013) provide further evidence of temporal causal binding from merely observed actions, and argue that causal binding receives a boost when the cause is perceived to be an intentional action. Their study provides an important methodological improvement over previous work because it offered better control over the perceptual stimuli. Moore et al. (2013) also provide fMRI data that suggests that the intentionality/causality interaction is subserved by similar brain regions as those involved in agency. Rohde and Ernst (2013) demonstrate that temporal adaptation is symmetrical. People adapt to action-outcome sequences such that the point of subjective simultaneity (PSS) of action and outcome shifts forward following exposure to action—delay—outcome sequences. Importantly, when—in a clever experimental setup—participants experienced outcome—delay—action sequences, the PSS analogously shifted backwards. While at first this might appear to violate the causal asymmetry, this result actually fits with the unity assumption inherent in Bayesian accounts of perception. Parsons et al. (2013) challenge an internal-clock based interpretation of temporal causal binding and instead make a convincing case for a realignment of the sensory and motor timeline. Asai and Kanayama (2012, 2013) conclude the volume with a contribution on the cutaneous rabbit effect (CRE), a tactile illusion resulting from a causal interpretation of spatio-temporal stimulation of the skin. Asai and Kanayama (2012, 2013) show that the CRE is modulated by visual stimuli, when these “fit” with the causal interpretation of the experienced spatio-temporal pattern.

In sum, this volume is testament to convergence of research on time perception and causal inference, in two ways: Firstly, as the two thematic blocks of articles show, there is now a clear recognition that Time and Causality mutually constrain each other in human experience. Not only do temporal parameters influence our causal experience, but the construal of causal relations in the mind also affects the way we perceive and experience time. Importantly, the volume also highlights the convergence of methods and disciplines that is happening in this area. Time and Causality are now firmly on the agenda of cognitive, developmental, social, clinical, and applied psychologists, perception researchers and psychophysicists, as well as neuroscientists and philosophers. Future questions include what exactly the relation is between time, causality, and agency, and to what extent they share common neural markers, how perceptual adaptation relates to the experience of agency, causality, and temporal order, and how extant models of time perception (i.e., internal clocks) relate to causality-induced shifts in time perception.

## References

[B6] Asai T. Kanayama N. (2012). “Cutaneous rabbit” hops toward a light: unimodal and cross-modal causality on the skin. Front. Psychol. 3:427 10.3389/fpsyg.2012.00427 23133432 PMC3490328

[B7] Asai T. Kanayama N. (2013). Erratum: Cutaneous rabbit “hops toward a light: unimodal and cross-modal causality on the skin”. Front. Psychol. 4:769 10.3389/fpsyg.2013.00769 24151484 PMC3799539

[B1] Buehner M. (2005). Contiguity and covariation in human causal inference. Learn. Behav. 33, 230–238 10.3758/BF03196065 16075841

[B2] Buehner M. May J. (2002). Knowledge mediates the timeframe of covariation assessment in human causal induction. Think. Reason. 8, 269–295 10.1080/13546780244000060

[B3] Dickinson A. (2001). Causal learning: association versus computation. Curr. Dir. Psychol. Sci. 10, 127–132 10.1111/1467-8721.00132

[B4] Einhorn H. J. Hogarth R. M. (1986). Judging probable cause. Psychol. Bull. 99, 3–19 10.1037/0033-2909.99.1.3

[B8] Faro D. McGill A. L. Hastie R. (2013). The influence of perceived causation on judgments of time: an integrative review and implications for decision-making. Front. Psychol. 4:217 10.3389/fpsyg.2013.00217 23717286 PMC3653058

[B9] Greville W. J. Buehner M. J. (2012). Assessing evidence for a common function of delay in causal learning and reward discounting. Front. Psychol. 3:460 10.3389/fpsyg.2012.00460 23162508 PMC3498961

[B5] Haggard P. Clark S. Kalogeras J. (2002). Voluntary action and conscious awareness. Nat. Neurosci. 5, 382–385 10.1038/nn827 11896397

[B10] Moore J. W. Teufel C. Subramaniam N. Davis G. Fletcher P. C. (2013). Attribution of intentional causation influences the perception of observed movements: behavioral evidence and neural correlates. Front. Psychol. 4:23 10.3389/fpsyg.2013.00023 23372562 PMC3557415

[B11] Msetfi R. M. Murphy R. A. Kornbrot D. E. (2012). Dysphoric mood states are related to sensitivity to temporal changes in contingency. Front. Psychol. 3:368 10.3389/fpsyg.2012.00368 23060837 PMC3459020

[B12] Parsons B. D. Novich S. D. Eagleman D. M. (2013). Motor-sensory recalibration modulates perceived simultaneity of cross-modal events at different distances. Front. Psychol. 4:46 10.3389/fpsyg.2013.00046 23549660 PMC3582016

[B13] Rankin M. L. McCormack T. (2013). The temporal priority principle: at what age does this develop? Front. Psychol. 4:178 10.3389/fpsyg.2013.00178 23658548 PMC3647108

[B14] Rohde M. Ernst M. O. (2013). To lead and to lag - forward and backward recalibration of perceived visuo-motor simultaneity. Front. Psychol. 3:599 10.3389/fpsyg.2012.00599 23346063 PMC3551234

[B15] Schlottmann A. Cole K. Watts R. White M. (2013). Domain-specific perceptual causality in children depends on the spatio-temporal configuration, not motion onset. Front. Psychol. 4:365 10.3389/fpsyg.2013.00365 23874308 PMC3708160

[B16] Woods A. J. Lehet M. Chatterjee A. (2012). Context modulates the contribution of time and space in causal inference. Front. Psychol. 3:371 10.3389/fpsyg.2012.00371 23162484 PMC3498891

